# Historical Inquiry into the State of Medicine among the Hebrews: Being an Inaugural Dissertation Maintained, by Meyer Lewin, under the Presidency of Professor Curtius Sprengel, on the 19th November, 1798, in the University of Halle

**Published:** 1799-07

**Authors:** 


### *Hiftorical Inquiry into the State of Medicine among the Hebrews: being an Inaugural Differtation maintained, by* MEYER LEWIN, *under the Prefidency of Profeffor* CURTIUS SPRENGEL, *on the 19th November,* 1798, *in the Univerfity of* HALLE.

SEVERAL authors have attempted to give an account of the hiftory of medicine among the Hebrews; but they in general attended more to inquiries relative to difeafes mentioned in Sacred Hiftory, than to the hiftory of the art itfelf. Few documents are to be found which relate to the medicine of the Hebrews, in thofe hiftories of medicine by REY, FRIEND, and BLACK; or even in Profeffor SPRENGEL's general hiftory of this fcience: fome articles are fcattered in his chapter on the medicine of the Egyptians, and in that on the medicine of the Eaftern nations.

The fects of Platonifts, and of Paracelfus, have, according to our author, derived their origin from Judaifm; and the Hebrews have always adopted the manners and cuftoms of other nations, notwithftanding the prohibitions laid on the people of Ifrael, againft imitating the manners of the barbarians. According to the doctrine of the Patriarchs, both good and evil came from GOD; invocation, or fubmiffion, were regarded as the only means of extirpating difeafes. When the Hebrews were in Egypt, they found there eftablifhed principles of medicine. By order of Jofeph, the Egyptian phyficians embalmed the body of Jacob. Mofes, who was acquainted with all the learning of the Egyptians, inferted that art in his code of medicinal and dietetic laws; he defcribed the white leprofy with great exactnefs: he alfo appears to have made ufe of their fuperftitious recipes, by the erection of the brazen ferpent. Already had he fuppreffed the Egyptian magicians, by prodigies more wonderful than theirs.

The tribe of Levi, at length affumed the practice of phyfic; the prophet attributed epidemic difeafes to heavenly wrath. The Philiftines, afflicted with leprous tumours, could not be cured till after the reftitution of the ark.

Even

Even the ſound of the harp of David, could allay the ravings of the maniac Saul. The ſcience of Solomon, who was perhaps inſtructed by the Phenecians, or the Arabs, was extended ſtill further. He was celebrated for his knowledge of plants and animals; and he alſo compoſed a treatiſe on the cure of diſeaſes, which was ſuppreſſed by Ezekias, leſt the ſacred remedies which made the ſacrifices of the tribe of Levi more praiſe-worthy, ſhould be abandoned for other remedies. Solomon alſo, if we may believe his writings, had the gift of aſſuaging diſeaſes by incantation, and of driving out devils by exorciſm, a practice which remained till the time of Joſephus the hiſtorian. The prophets then aſſumed the practice of phyſic. The hand of Jeroboam was cured by a prophet, whom he had offended and Elias reſtored the ſon of the widow of Zarpatha. Eliſha recovered the ſon of a Shunamite, and Naman cured a Syrian chief of the leproſy, by ordering him to be immerſed in the river Jordan; Iſaiah alſo reſtored Ezekias to life, by applying to his tumours a cataplaſm of figs. About the year 640, before the Chriſtian era, the Hebrews were diſperſed in Medea and Aſſyria, and ſubmitted to the king of Babylon. This was the epoch in which the religion of ZERDUSCH or ZOROASTER, began to ſpread. The exiled Hebrews combined many of his dogmas with thoſe of their religion; this gave riſe to the Kabbale, a ſort of Eaſtern philoſophy, which the Alexandrians at length combined with the ſuperſtitions of the Greeks. It was now that medicine began to aſſume another form. Among the good Genii, was one who watched over health; One of theſe was called BOETE: he cauſed diſeaſes, another named MAZDEJESNOIS, was empowered to try remedies on the people who were under the influence of the Dews, or evil demons.

During the reign of Cyrus, who ſubdued the kingdom of Babylon, the Greeks were enabled to learn the doctrine of Zoroaſter, and appropriate it to their own uſes. But this theory was not entirely derived from Zoroaſter; it owed its origin to a more ancient prophet, named Ham; the author of this philoſophy having collected its principles from among the Indians and Bramins: this account is affirmed by Profeſſor Sprengel, not according to the futile arguments of WILFORD, or the ridiculous and contemptible calculations of JONES and KLEUKER, but from venerable monuments, from books which ſurpaſs in antiquity any which we poſſeſs, and from the moſt exact aſtronomical calculations. The analogy of the ſyſtem of the Bramins to that of Zoroaſter, and the perſpicuity which it has given to their ideas, clearly prove that it has paſſed from India to Media, and that it was not tranſported by Zoroaſter and his diſciples to the banks of the Indus. It teaches us that GOD is eternal; that he produced, before the moſt diſtant ages, three perſons or ſubſtances, united in one, that is to ſay, earth, water, and fire, from from which the whole army of angels *(dewta)* were created. The Indians reckon ſeven worlds, which are under the influence of either the good or evil principle: they adore the Sun, as being the ſymbol of good emanations; man, born on the good principle, if the ſoul be conſidered, but on the bad principle, as to what relates to the body, is encloſed in his own body that he may ſuffer pain; and when this body has undergone ſufficient chaſtiſement, the man becomes more pure, and approaches nearer to perfection. The Samaritans who, in a life of ſolitude and retirement, inceſſantly contemplating the deity, abſtained from the uſe of fleſh. Such, in ancient times, were the true Indian Phyſicians.

This Oriental doctrine was tranſplanted into Media by Zoroaſter, and from thence, in the time of Cyrus, to Perſia, where the exiled Jews, who were without temples and ſacrifices, adopted an auſtere and contemplative life: thence it was, that MAGASTHENES, who lived in the time of SELEUCUS NICATOR, has confounded the religion of the Indians with that of the Hebrews.

Amongſt the principal tenets which the Hebrews have derived from Babylon, is that of the immortality of the ſoul; of which they do not appear to have had any previous information, and of which the firſt traces are to be found in the ſubſequent apocryphal books of DANIEL hence originated their; belief in the power of angels and demons, to produced or cure diſeaſes.

At this period it was again neceſſary to reſume the occult doctrine of the *myſtic word of God*, which is regarded as a perſonal illumination: this dogma aroſe from the philoſophy of Zoroaſter, and was thence introduced into the Jewiſh ſchools.

The Hebrew people, by their repeated captivity, had loſt the knowledge of their primitive language, and could no longer read their laws in the Hebrew tongue. By order of ESDRAS, a Chaldaic paraphraſe was corrected, for the purpoſe of being read in the ſynagogue. Thus it appears that Eſdras was the firſt who introduced the uſe of the Chaldaic language. At this period, MANASSES, chief of the ſect of Samaritans erected a temple upon Mount Garizim, in which he eſtabliſhed a religion, of the knowledge of which we have been almoſt deprived by the lapſe of time: this was the origin of the Eſſians and Samaritans. The Jews who, in the reign of JEREMIAH, had fled into Egypt, as well as thoſe who were led thither by ARTAXERXES III. and PTOLEMY LAGUS, had a greater partiality for the philoſophic doctrine of the Greeks of Alexandria than for the degenerated ſyſtem of the Platoniſts; the ſophiſtry of the Alexandrians, their love of paradox, and their taſte for divinity, were ſimilar to their Oriental philoſophy.

The latter sect derived its name from a Syrian word, which signifies *saint*: its first origin is discovered to have been in the time of JUDAS MACCABEUS. It is supposed, that the Essians of Syria, and those of Egypt, differed greatly in their opinions: the former followed more particularly the Eastern philosophy, and the latter that of Alexandria. These last were nominated *Therapeutians*, because they led a life of experiment and contemplation.

The Essians made a vow to respect their books as much as the names of angels. PHILO, who calls the second angel the great Physician, does not deviate in any considerable degree from the system of the Essians.

This contemplative life of the Essians made a forcible impression on the minds of some, who, possessed by a religious phrenzy, pretended to be deranged in their intellects, until they had obtained what they wished for.— These enthusiasts met together frequently in Egypt; they abandoned their parents, their children, and their property; and lived in villages, assiduously reading the works of the prophets, so that even in their dreams, their imagination presented nothing but divine objects; they prayed twice every day; they never ate till after sun set, devoting the day to the study of wisdom, the night to the refection, and repose of the body:—their food was only bread, with salt, and hysop; they religiously set apart the seventh day, when they met together at a frugal banquet, which was spread on leaves of the papyrus.

The Author says, in another place, that there were of this sect in Egypt four thousand, who never killed any animal, who avoided cities, rejected the arts and sciences, and contemned riches, but inculcated benevolence and hospitality; insomuch that at length their manners became so pure, that the most cruel tyrants could never accuse them of the smallest crime.

The Essians of Syria adopted principles rather different; acording to them the soul, naturally immortal, was created to practice justice. They celebrated their festivals in common temples, and cultivated their fields without the aid of slaves, by mutually assisting each other: they selected the most exemplary for priests, who acted as collectors, and prepared their necessaries, they abstained from oaths, practised sobriety, justice, and the religion of GOD; they were always habited in white, and kept continual silence for a year together, which rendered them similar to the Pythagoreans. They were looked upon as the primitive Christians; it is certain, at least, that they were the disciples of Moses, who had collected from the sacred writings of the Greeks, and Orientals, every thing that could excite the mind, and inspire a fanatical enthusiasm.

[Want of room will not permit us to extend the account of this article.]

